# Down-regulation of the tumour suppressor κ-opioid receptor predicts poor prognosis in hepatocellular carcinoma patients

**DOI:** 10.1186/s12885-017-3541-9

**Published:** 2017-08-18

**Authors:** Dongtai Chen, Yonghua Chen, Yan Yan, Jiahao Pan, Wei Xing, Qiang Li, Weian Zeng

**Affiliations:** 1 Department of Anesthesiology, Sun Yat-sen University Cancer Center, State Key Laboratory of Oncology in South China, Collaborative Innovation Center for Cancer Medicine, No. 651, Dongfeng Road East, Guangzhou, Guangdong 510060 China; 2 Department of Anesthesiology, HuiZhou Municipal Central Hospital, Huizhou, China

**Keywords:** Hepatocellular carcinoma, κ-opioid receptor, Prognosis, Tumour suppressor

## Abstract

**Background:**

Opioid receptors have become increasingly implicated in cancer progression and long-term patient outcomes. However, the expression and significance of the κ-opioid receptor (KOR) in hepatocellular carcinoma (HCC) remain unclear.

**Methods:**

In this study, KOR mRNA expression was analysed by real-time quantitative PCR in 64 pairs of HCC tumour tissues and adjacent non-tumour tissues, and KOR protein expression was analysed by immunohistochemistry in 174 HCC patients. We investigated the correlation between KOR expression and clinicopathological parameters to illustrate the potential prognostic significance of KOR expression in HCC.

**Results:**

KOR mRNA expression was significantly down-regulated in 79.69% (51 of 64) of the HCC tumour samples, and KOR expression in tumour tissue was significantly lower than that in adjacent non-tumour tissues (*P* < 0.001). ROC curve analysis showed that KOR mRNA expression yielded AUC of 0.745, for the detection of HCC patients. Low KOR mRNA expression in HCC was correlated with aggressive clinicopathological parameters, such as tumour size (*P* = 0.015), differentiation grade (*P* = 0.011), and TNM stage (*P* = 0.021). Moreover, down-regulation of KOR protein expression in HCC tissues was detected in 174 HCC patients. Similarly, negative KOR protein expression was significantly correlated with aggressive clinicopathological features, such as tumour size (*P* = 0.002), vascular invasion (*P* = 0.003), differentiation grade (*P* = 0.026), and TNM stage (*P* = 0.030). Furthermore, Kaplan-Meier survival analysis demonstrated that down-regulation of KOR in HCC indicated poor prognosis. KOR deficiency (KOR^T < N^) was correlated to a shorter survival rate and an increased recurrence (both *P* < 0.001). In univariate and multivariate survival analyses, KOR was identified as a promising independent risk factor for both overall survival (OS, both *P* < 0.001) and recurrence-free survival (RFS, both *P* < 0.001).

**Conclusions:**

Down-regulation of KOR in HCC tumour tissues has a strong association with poor prognosis and KOR might be a potential tumour suppressor.

**Electronic supplementary material:**

The online version of this article (doi:10.1186/s12885-017-3541-9) contains supplementary material, which is available to authorized users.

## Background

Hepatocellular carcinoma (HCC), as a common malignancy, has an increasing incidence and mortality globally, especially in Asia [1]. Worldwide, there are more than 50% of the cases in China alone, according to the epidemiologic report [2]. This cancer has a very poor survival rate even with advanced diagnostic strategies and improved therapies. The prognosis for HCC after resection is still discouraging due to the potential for residual tumor and the high rate of tumor recurrence, which exceeds 60% [3]. Therefore, investing a valuable biomarker to better evaluate the diagnosis and prognosis of HCC patients will be beneficial in the guidance of treatment and inhibition of metastasis.

Opioids are wildly used in pain management of cancer patients, no doubt that interest in the possibility of opioids may influence the course of cancer development is not recent. Opioids have been shown to accelerate the growth of tumour cells and induce metastasis [4, 5], whereas other studies have reported that opioids can induce apoptosis in several cancer cells, such as lung cancer, colon cancer and breast cancer [6–9]. Opioid receptors, with opioids as ligands, belong to a group of G protein-coupled receptors [10]. In general, opioid receptors contain three subtypes, μ, κ, and δ (MOR, KOR and DOR, respectively), which modulate a variety of physiological functions such as pain regulation, emotional tone, and cognitive functions [11]. Opioid receptors were discovered both in neural tissues (brain and spinal cord) and a wide spectrum of peripheral extraneural tissues (spleen, stomach, lung, pancreas, liver, heart, blood, and blood vessels) [12]. The expression profile of opioid receptors in different cancer cells has also been reported [13] and experimental studies in investing the effects of opioid receptor agonists and antagonists on the proliferation and metastasis of cancer both in vivo and in vitro study have received lots of attention. Morphine, a MOR agonist, were shown to possess antitumor effects [6]. In contrast, other reports described tumor-promoting effects of morphine by immunosuppression [14] or inducing angiogenesis [15].

Although the reports in the literature are inconsistent, opioid receptors expressed in tumour cells may have an implication in tumour progression [16, 17]. KOR expression has been reported in various cancer cells, such as small lung cancer cell and oesophageal cancer cell [18, 19]. Furthermore, KOR expression is up-regulated in esophageal squamous cell carcinoma (ESCC) tissues and patients with an elevated nuclear KOR expression in ESCC have a worse prognosis [19]. In contrast, an in vivo assay revealed that xenograft tumors in KOR knock-out mice demonstrated increased tumour growth and promoted angiogenesis [20]. Therefore, the effect of KOR expression in different cancers is variable. The potential role of KOR in HCC progression, including recurrence and metastasis, is unknown. In this research, we aimed to detect the clinical significance of the expression of KOR in HCC patients and investigate the potential effects of KOR expression on patient prognosis.

## Methods

### HCC patients and tissue specimens

We got an approval from Committee for Ethical Review of Research at Sun Yat-sen University Cancer Center. All paraffin-embedded tissues were collected from 174 patients who had undergone curative resection for primary HCC between 2003 and 2006 at Sun Yat-sen University Cancer Center (Guangzhou, China). The inclusion criteria were a distinct pathological diagnosis with an absence of anticancer therapy prior to surgical resection or distant metastasis, and the availability of follow-up data. All 174 pairs of primary HCC tissue samples and adjacent non-tumour tissues were used for immunohistochemical analysis. In addition, 64 pairs of fresh liver tumour tissues and adjacent non-neoplastic tissues were collected instantly after surgical resection during May and July in 2016 and stored in liquid nitrogen. The samples were later analysed with quantitative real-time PCR analysis.

### Follow up

The follow-up data were summarized at the end of January 2015, and the median follow-up time was 56.5 months. One hundred forty eight males and twenty six females were collected in our study, and the median age was 50 years. Recurrences were confirmed by serum a-fetoprotein (AFP) levels, abdomen ultrasound every 2 months, and computed tomography (CT) or magnetic resonance imaging (MRI) every 6 months after hepatectomy. All follow-up data were collected by outpatient visit and telephone follow-up. We classified the tumour-node-metastasis (TNM) stage according to the 6th edition Cancer/International Union Against Cancer staging system by American Joint Committee (2002). The tumour differentiation grade was defined according to the Edmondson-Steiner grading system. Recurrence-free survival (RFS) and overall survival (OS) were the primary endpoints. The definition of RFS was the interval between surgery and recurrence or from the time of surgery to the last observation collected. The definition of OS was the period from the date of resection to the endpoint of survival or the endpoint of the follow-up appointment.

### Real-time quantitative PCR analysis

TRIzol reagent (Gibco Invitrogen, Carlsbad, USA) was used to extract total RNA, and a PrimeScript RT Kit (TaKaRa, Japan) was used to performed reverse transcription. For real-time quantitative PCR analysis, we used SYBR Green qPCR SuperMix (Gibco Invitrogen), and the CFX96™ Real-Time PCR Detection System (Bio-Rad, USA). GAPDH expression was used as an internal control. Here were the primers we used: KOR, forward, 5′-CGTCTGCTACACCCTGATGATC-3′, reverse, 5′-CTCTCGGGAGCCAGAAAGG’; GAPDH, forward, 5′-AGAAGGCTGGGGCTCATTTG-3′, reverse, 5′-AGGGGCCATCCACAGTCTTC-3′.

### Immunohistochemical analysis of KOR

We cut paraffin-embedded tissue samples into 4-μm sections and used for immunohistochemistry (IHC). Briefly, the tissue samples were deparaffinised, rehydrated and blocked with 10% normal goat serum, as the procedure we used in previous studies [21]. Then, we incubated the samples with anti-KOR primary antibody (R&D Systems, Minneapolis, USA) at 4 degree Celsius overnight. Afterwards, EnVision kit (Dako Cytomation, Carpinteria, USA) was used to detect antibodies of tissues sections. We graded the samples according to the staining intensity and percentage of cells stained with a score of 0–3. According to the staining intensity, we classified KOR protein staining as follows: 0 = absent expression, 1 = weak expression, 2 = moderate expression, 3 = strong expression. In addition, we determined the percentage of positive tumour cells staining with a score of 0–100. The two scores were multiplied to produce a weighted score for each case. Theoretically, the scores ranged from 0 (0% of cells staining) to 3 (100 × 3/100). Furthermore, we characterized a score of ≤1 as “KOR-negative” and a score of >1 as “KOR-positive”. IHC assessments were carried by two experienced pathologists in a double-blind manner. The IHC pictures were captured with an Olympus BX41 microscope (Olympus Optical, Japan) at 200 magnifications.

### Statistical analysis

All data in this study were evaluated with the SPSS 16.0 software (SPSS Inc., Chicago, USA). The results of real-time quantitative PCR were determined using Student’s t test. Survival analysis was demonstrated using the Kaplan–Meier method and log-rank tests. The relevance between KOR expression and clinicopathological parameters was carried out using the chi-square test. The Cox proportional hazards regression model was constructed to evaluate prognostic factors by univariate and multivariate analyses. Differences were considered statistically significant at a value of *P* < 0.05.

## Results

### KOR is down-regulated in human HCC

**Fig. 1:**
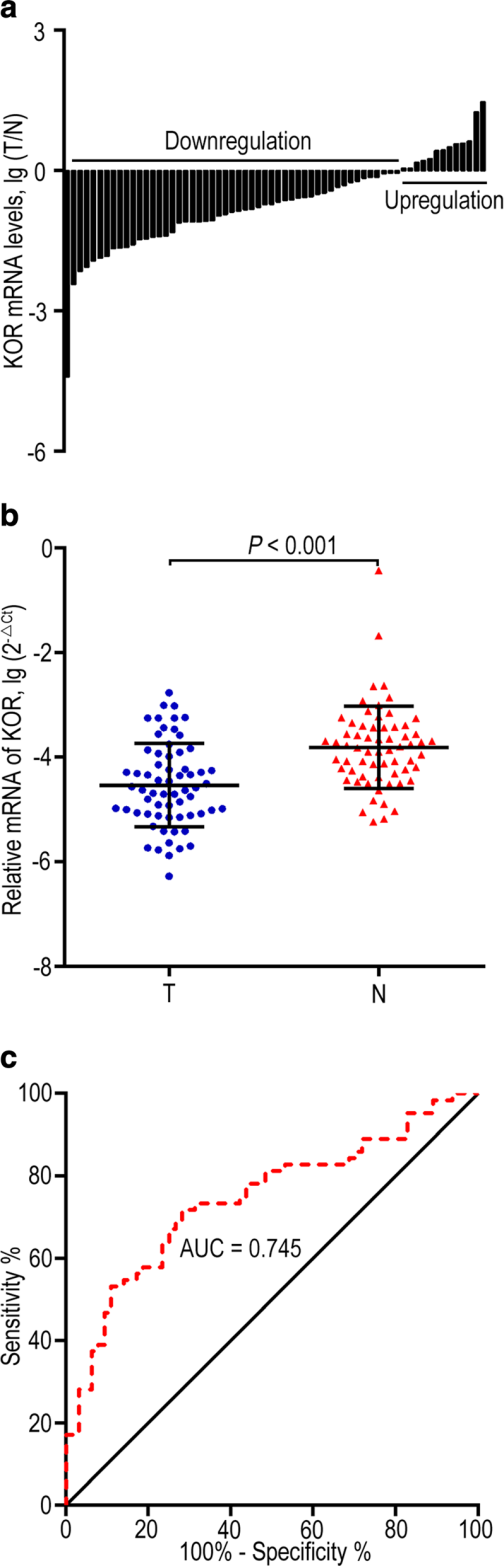
Down-regulation of KOR mRNA in HCC. **a** KOR mRNA expression in 64 paired HCC tumour tissues (T) and corresponding non-tumour tissues (N). The data revealed that down-regulation of KOR was detected in 79.69% (51 of 64) of HCC samples. **b** Relative expression levels of KOR in tumour tissues are significantly lower than in corresponding non-tumour tissues (*P* < 0.001). **c** ROC curve was constructed according to KOR mRNA expression in HCC and adjacent non-tumour tissues

To detect the potential role of KOR in HCC, real-time quantitative PCR was conducted to compare the mRNA expression level of KOR in 64 pairs of tumour tissues and adjacent non-tumour tissues. We detected a down-regulation of KOR in 79.69% (51 of 64) of the HCC samples (Fig. 1a); KOR expression was significantly lower than in adjacent non-neoplastic tissues (*P* < 0.001; Fig. 1b). Meanwhile, a receiver operating characteristic (ROC) curve was constructed and the result showed that KOR mRNA expression yielded area under curve (AUC) of 0.745, for the detection of HCC patients (Fig. 1c).

**Fig. 2:**
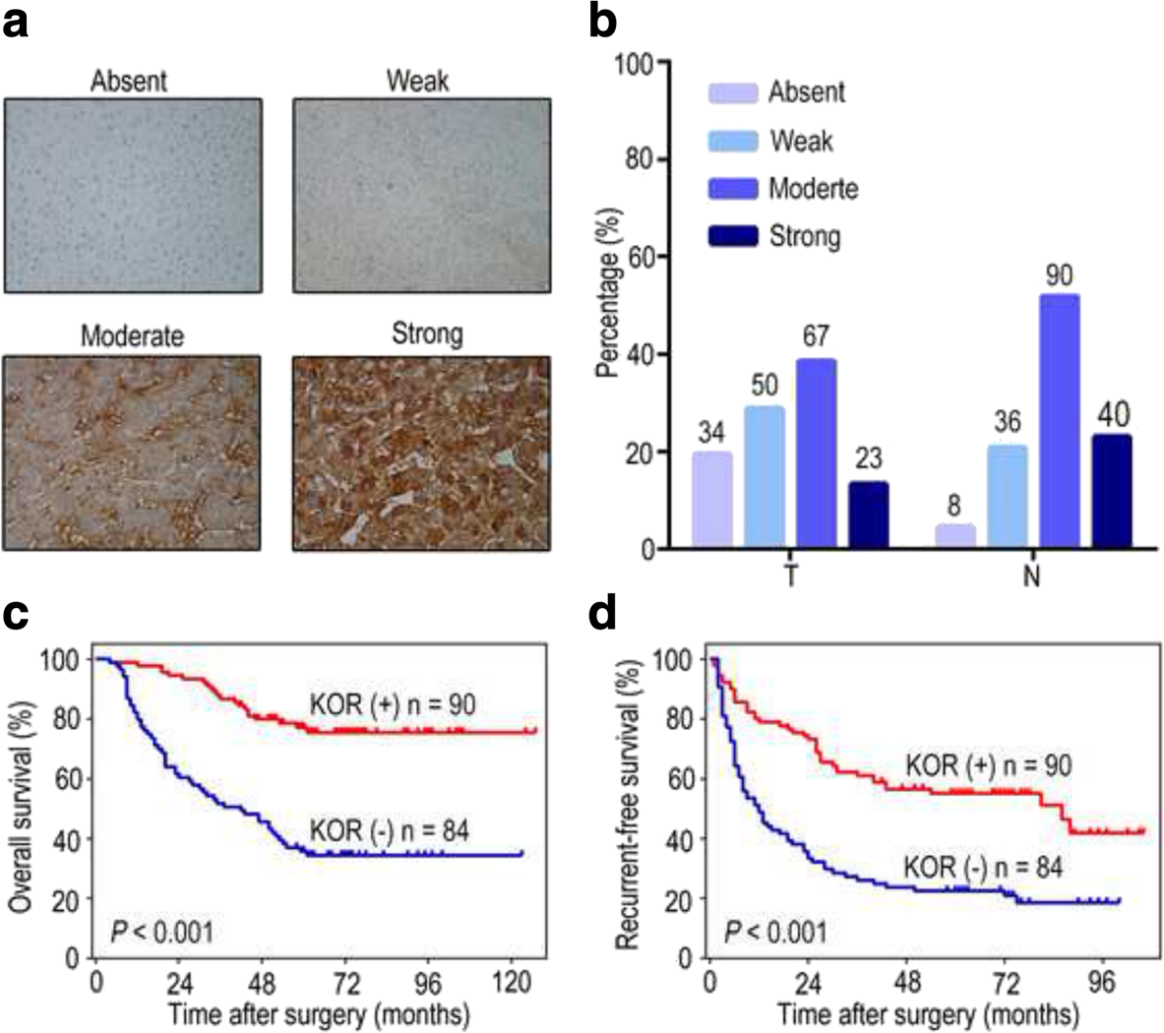
Down-regulation of KOR protein in HCC. **a** Representative immunohistochemical staining of KOR protein expression in 174 HCC tissue samples (magnification ×200). Absent or weak staining was defined as negative expression, while moderate or strong staining was defined as positive expression. **b** The presence of KOR staining in HCC tissues and corresponding non-tumour tissues. **c**, **d** Kaplan-Meier analysis for overall survival (OS) and recurrence-free survival (RFS) of 174 HCC patients in correlation with KOR expression. The OS and RFS rates were significantly decreased in KOR-negative HCC patients compared with those in the KOR-positive group (both *P* < 0.001). The duplicated images in Figs. 2, 3 and Additional file 1: Figure S1 represent the same experiment

The staining pattern of KOR protein expression in HCC tissue and corresponding adjacent non-tumour tissue was frequently observed (Additional file 1: Figure S1). According to the staining results, we classified the KOR expression in tumour tissues as negative (absent and weak staining) or positive (moderate and strong staining) (Fig. 2a). The results showed that KOR protein expression was positive in 51.7% (90 of 174: moderate, *n* = 67; strong, *n* = 23) of the tumour tissues and in 74.7% (130 of 174: moderate, *n* = 90; strong, *n* = 40) of the corresponding non-tumour tissues (Fig. 2b).

### Low KOR expression correlates with aggressive clinicopathological parameters in patients with HCC

To better elucidate the clinical relevance of KOR expression in HCC, we investigated the correlation of clinicopathological parameters with KOR expression in HCC samples (KOR mRNA and protein expression, respectively). Interestingly, low KOR mRNA expression in HCC was correlated with aggressive clinicopathological parameters, such as tumour size (*P* = 0.015), differentiation grade (*P* = 0.011), and TNM stage (*P* = 0.021, Table 1). Similar results were found in the relationship between KOR-negative protein expression and aggressive clinicopathological features, such as tumour size (*P* = 0.002), vascular invasion (*P* = 0.003), differentiation grade (*P* = 0.026), and TNM stage (*P* = 0.030; Table 2).

**Table 1:** Correlation of KOR mRNA expression with clinicopathological features in HCC (*n* = 64)

Variables	Cases	KOR mRNA		
		T < N (*n* = 51)	T ≥ N (*n* = 13)	*P*
Gender				
Female	8	5	3	0.196
Male	56	46	10	
Age, years				
≤ 50	28	22	6	0.845
> 50	36	29	7	
HBsAg				
Negative	8	6	2	0.725
Positive	56	45	11	
Child-Pugh classification^a^				
A	61	49	12	0.566
B	3	2	1	
Serum AFP, ng/ml				
≤ 20	30	21	9	0.070
> 20	34	30	4	
Tumor Number				
Single	48	37	11	0.370
Multiple	16	14	2	
Tumor size, cm				
≤ 5	35	24	11	0.015*
> 5	29	27	2	
Tumor capsule				
No/incomplete	31	23	8	0.290
Yes	33	28	5	
Vascular invasion				
No	58	46	12	0.816
Yes	6	5	1	
Liver cirrhosis				
Absent	11	7	4	0.146
Present	53	44	9	
Differentiation grade				
I / II	34	23	11	0.011*
III/IV	30	28	2	
TNM stage				
I	36	25	11	0.021*
II/III	28	26	2	

The data are reported as number. *P*-values were obtained from the chi-square test. ^a^No patient with Child-Pugh class C was found. *Statistical significance was set to *P* < 0.05

**Table 2:** Correlation of KOR protein expression with clinicopathological features in HCC (*n* = 174)

Variables	Cases	KOR protein		
		High expression (*n* = 182)	Low expression (*n* = 126)	*P*
Gender				
Female	37	22	15	0.509
Male	271	160	111	
Age, years				
≤ 50	93	47	46	0.737
> 50	81	43	38	
HBsAg				
Negative	23	13	10	0.621
Positive	151	77	74	
Child-Pugh classification^a^				
A	167	81	86	0.770
B	7	3	4	
Serum AFP, ng/ml				
≤ 20	55	34	21	0.070
> 20	119	56	63	
Tumor Number				
Single	143	75	68	0.682
Multiple	31	15	16	
Tumor size, cm				
≤ 5	71	47	24	0.002*
> 5	103	43	60	
Tumor capsule				
No/incomplete	77	35	42	0.140
Yes	97	55	42	
Vascular invasion				
No	163	89	74	0.003*
Yes	11	1	10	
Liver cirrhosis				
Absent	32	19	13	0.338
Present	142	71	71	
Differentiation grade				
I / II	102	60	42	0.026*
III/IV	72	30	42	
TNM stage				
I	129	73	56	0.030*
II/III	45	17	28	

The data are reported as number. *P*-values were obtained from the chi-square test. ^a^No patient with Child-Pugh class C was found. *Statistical significance was set to *P* < 0.05

### Down-regulation of KOR indicates poor prognosis in HCC patients

Based on the complete follow-up data from the entire study population, the RFS and OS rates were 43 and 68% at 3 years and 39 and 55% at 5 years, respectively. To confirm the correlation between the KOR expression levels in tumour tissues and HCC prognosis, we compared time to RFS and OS in the KOR-positive and KOR-negative groups. Kaplan-Meier survival analysis displayed that patients with HCC in the KOR-negative group had worse RFS and OS than did those in the KOR-positive group. The five-year rates of RFS and OS were 23 and 34% in the KOR-negative group compared to 55 and 76% in the KOR-positive group, respectively (both *P* < 0.001; Fig. 2c).

**Fig. 3:**
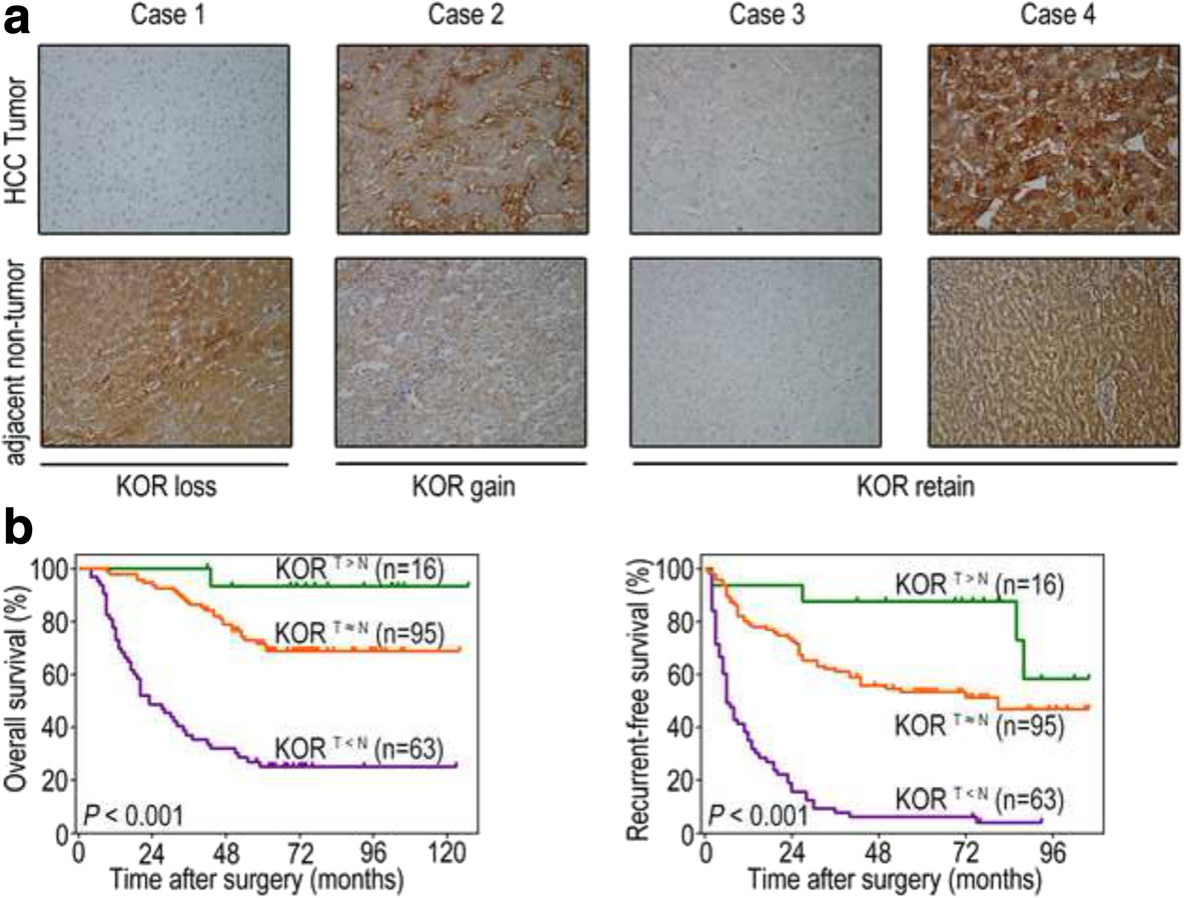
Down-regulation of KOR correlates with poor prognosis in HCC patients. **a** Representative immunostaining images of KOR loss/gain/retain cases in HCC tumour tissues and adjacent non-tumour tissues (magnification ×200). **b** Kaplan-Meier curves for OS and RFS according to KOR expression and KOR loss/gain/retain in the validation cohort. The duplicated images in Figs. 2, 3 and Additional file 1: Figure S1 represent the same experiment

We further classified the patients into three groups according to the intensity of IHC staining in tumour tissues and adjacent non-tumour tissue, i.e. the KOR loss group (KOR^T < N^), KOR gain group (KOR^T > N^), and KOR retain group (KOR^T ≈ N^), to evaluate the significance of KOR loss and gain in tumours compared to adjacent non-tumour tissues (Fig. 3a). Consist with our findings thus far, the KOR loss group (*n* = 63) exhibited the shortest OS rate (median, 23 months) and RFS rate (median, 6 months), whereas the KOR gain group (*n* = 16) exhibited the best survival rates (OS median: 79 months; RFS median: 75 months); the KOR retain group (*n* = 95) ranked in the middle in terms of survival (OS median: 61 months; RFS median: 50 months; Fig. 3b).

### Down-regulation of KOR in HCC is an independent prognostic factor

To determine whether the negative KOR expression in tumour tissues was an independent prognostic factor for HCC, univariate and multivariate survival analyses were conducted. In the results from the univariate analysis, the down-regulation of KOR (*P* < 0.001), hepatitis B surface antigen (HBsAg, *P* = 0.028), tumour size (*P* < 0.001), TNM stage (*P* = 0.004) were negative prognostic factors for OS in HCC patients. However, the down-regulation of KOR (*P* < 0.001), HBsAg (*P* = 0.008), tumour size (*P* = 0.002), differentiation grade (*P* = 0.036) were significantly linked to poor RFS rates in HCC patients. According to multivariate Cox regression analysis, the down-regulation of KOR (*P* < 0.001) and tumour size (*P* = 0.017) were identified as independent risk factors for OS, while the down-regulation of KOR (*P* < 0.001), tumour size (*P* = 0.043) and HBsAg (*P* = 0.014) were recognized as independent risk factors for RFS. The hazard ratios of low KOR expression for OS and RFS were 0.526 (95% CI, 0.408–0.679) and 0.669 (95% CI, 0.543–0.810), respectively (Tables 3, 4).

**Table 3:** Univariate and multivariate analysis of factors associated with survival in HCC patients (*n* = 174)

	OS					
	Univariate analysis			Multivariate analysis		
	HR	95% CI	*P*	HR	95% CI	*P*
Gender (Female vs. Male)	0.755	0.415–1.374	0.354			
Age, years (≤ 50 vs. > 50)	1.382	0.878–2.177	0.159			
HBsAg (Negative vs. Positive)	2.922	1.067–8.003	0.028*	2.729	0.994–7.491	0.051
Child-Pugh classification (A vs. B)	1.669	0.609–4.577	0.320			
Serum AFP, ng/ml (≤ 20 vs. > 20)	1.133	0.689–1.863	0.62			
Tumor Number (Single vs. Multiple)	1.638	0.953–2.814	0.07			
Tumor size, cm (≤ 5 vs. > 5)	2.429	1.455–4.056	< 0.001*	1.888	1.121–3.179	0.017*
Tumor capsule (No/ incomplete vs. Yes)	0.662	0.421–1.042	0.072			
Vascular invasion (N0 vs. Yes)	2.126	0.976–4.634	0.051			
Liver cirrhosis (Absent vs. Present)	1.097	0.592–2.035	0.767			
Differentiation grade (I / II vs. III/IV)	1.348	0.856–2.125	0.194			
TNM stage (I vs. II/III)	1.968	1.222–3.171	0.004*	1.558	0.961–2.528	0.072
KOR (Positive vs. Negative)	0.499	0.387–0.642	< 0.001*	0.526	0.408–0.679	< 0.001*

*P*-values were obtained from the Cox proportional hazards regression analysis

*Statistical significance was set to *P* < 0.05

**Table 4:** Univariate and multivariate analysis of factors associated with recurrence in HCC patients (*n* = 174)

	RFS					
	Univariate analysis			Multivariate analysis		
	HR	95% CI	*P*	HR	95% CI	*P*
Gender (Female vs. Male)	0.816	0.492–1.352	0.422			
Age, years (≤ 50 vs. > 50)	1.342	0.922–1.953	0.117			
HBsAg (Negative vs. Positive)	2.518	1.225–5.176	0.008*	2.580	1.252–5.305	0.014*
Child-Pugh classification (A vs. B)	1.824	0.799–4.165	0.154			
Serum AFP, ng/ml (≤ 20 vs. > 20)	1.103	0.738–1.648	0.627			
Tumor Number (Single vs. Multiple)	1.333	0.827–2.148	0.229			
Tumor size, cm (≤ 5 vs. > 5)	1.841	1.236–2.740	0.002*	1.530	1.013–2.306	0.043*
Tumor capsule (No/ incomplete vs. Yes)	0.78	0.535–1.136	0.187			
Vascular invasion (N0 vs. Yes)	1.706	0.828–3.515	0.136			
Liver cirrhosis (Absent vs. Present)	1.109	0.669–1.839	0.682			
Differentiation grade (I / II vs. III/IV)	1.481	1.017–2.158	0.036*	1.224	0.823–1.791	0.327
TNM stage (I vs. II/III)	1.479	0.975–2.244	0.06			
KOR (Positive vs. Negative)	0.626	0.516–0.760	< 0.001*	0.669	0.543–0.810	< 0.001*

*P*-values were obtained from the Cox proportional hazards regression analysis

*Statistical significance was set to *P* < 0.05

**Fig. 4:**
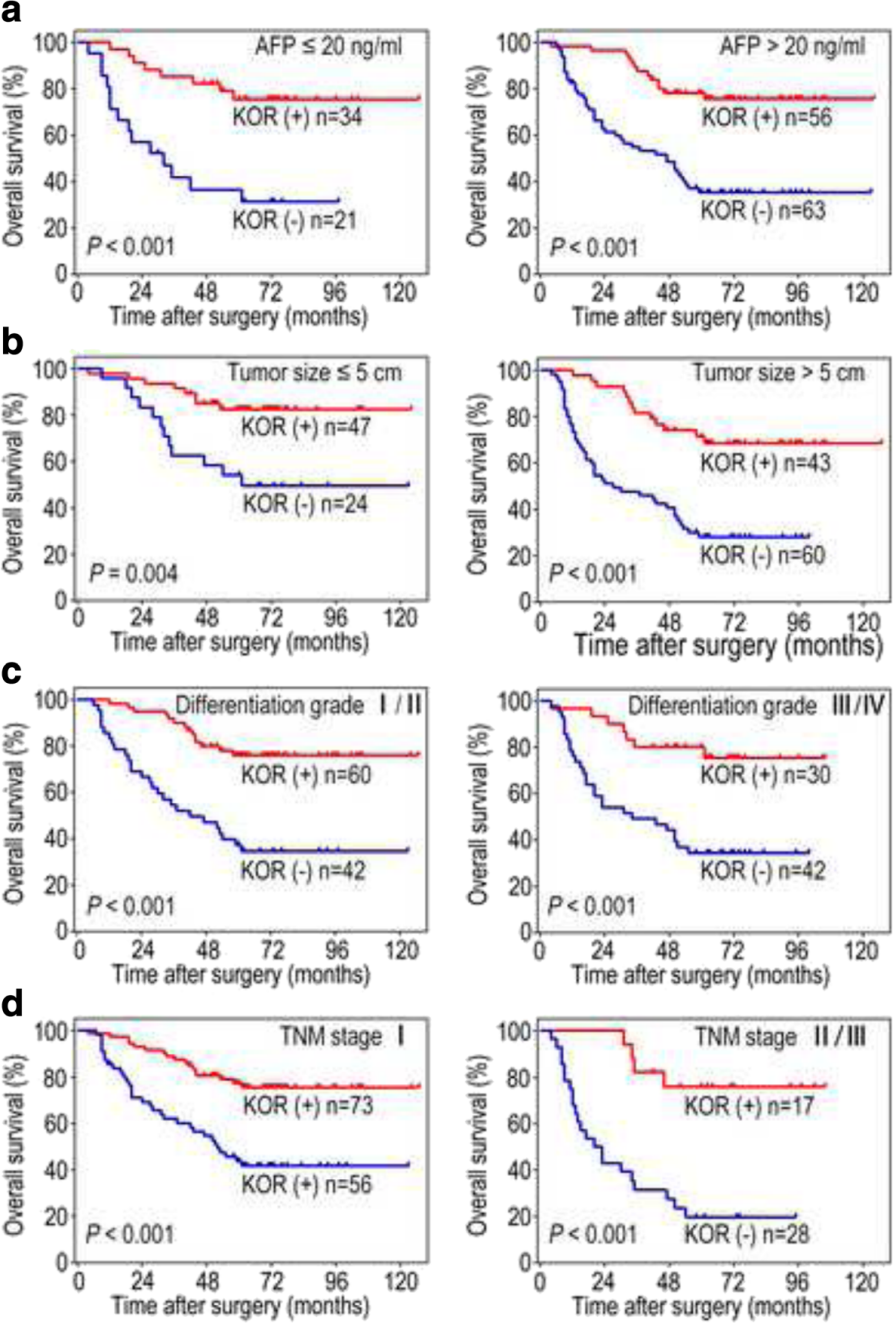
KOR-positive HCC patients in different subgroups showed a better OS than did KOR-negative HCC patients. **a** The cohort was classified into 2 groups based on AFP levels with a cut-off point of 20 ng/ml: AFP ≤ 20 ng/ml and AFP > 20 ng/ml; **b** The cohort was classified into 2 groups based on tumour size with a cut-off point of 5 cm: Tumour size ≤5 cm and Tumour size >5 cm; **c** The cohort was classified into 2 groups based on differentiation grade: Differentiation grade I/II and Differentiation grade III/IV; **d** The cohort was classified into 2 groups based on TNM stage: TNM stage I and TNM stage II/III

**Fig. 5:**
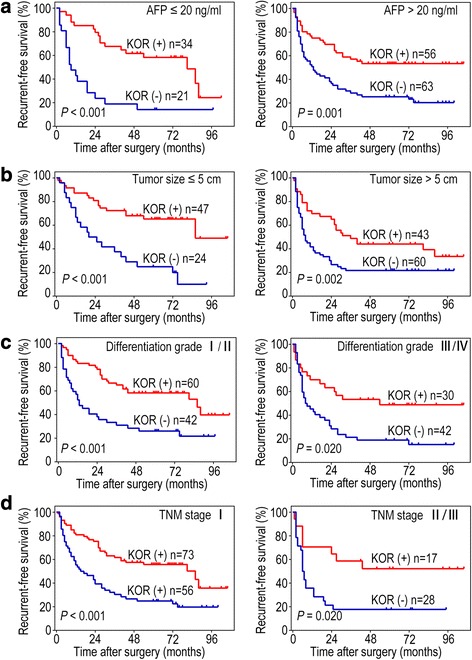
KOR-positive HCC patients in different subgroups showed a better RFS than the KOR-negative HCC patients. **a** The cohort was classified into 2 groups based on AFP levels with a cut-off point of 20 ng/ml: AFP ≤ 20 ng/ml and AFP > 20 ng/ml; **b** The cohort was classified into 2 groups based on tumour size with a cut-off point of 5 cm: Tumour size ≤5 cm and Tumour size >5 cm; **c** The cohort was classified into 2 groups based on differentiation grade: Differentiation grade I/II and Differentiation grade III/IV; **d** The cohort was classified into 2 groups based on TNM stage: TNM stage I and TNM stage II/III

To further detect the prognostic value of KOR, patients were divided into 4 subgroups: (1) Alpha–fetoprotein (AFP) ≤ 20 ng/ml and AFP > 20 ng/ml; (2) Tumour size ≤5 cm and Tumour size >5 cm; (3) Differentiation grade I/II and Differentiation grade III/IV; and (4) TNM stage I and TNM stage II/III. Patients in the KOR-positive group exhibited a significantly better OS and RFS than did those in the KOR-negative group, regardless of subgroup (all *P* < 0.05; Figs. 4, 5).

## Discussion

Patients with HCC, which is the most prevalent malignant carcinoma, exhibit a poor outcome, even after curative resection [1, 22]. As previous reports have shown, the 5-year recurrence rate of HCC patients ranges from 50 to 70% [2]. In our cohort study, the 5-year recurrence rate of HCC patients was 61%. Therefore, dependable tumour biomarkers are urgently needed to help identify patients who are at a high risk of poor survival and to establish individualized treatment programmes.

The reports showed that opioids administration is one of the factors that could influence tumour progression [23–25]. Opioid receptors (MOR, KOR, and DOR) are the targets of opioids and are classified into the superfamily of G-protein-coupled receptors, which modulate pain and emotional regulation [12]. Opioid receptors are expressed in many tumours and are implicated in cell proliferation and metastasis. DOR and MOR are overexpressed in lung cancer [26, 27], and the overexpression of MOR enhances cancer progression by regulating Epidermal Growth Factor (EGF)-induced signalling events [28] and epithelial mesenchymal transition (EMT) events [17]. In addition, previous studies have demonstrated that KOR takes part in the tumourigenesis and progression of ESCC [19] but that the activation of KOR inhibits the growth of lung cancer cells [18]. These findings indicate that KOR expression plays different roles in various carcinomas. Nevertheless, the expression status of KOR in HCC remains unknown.

In this study, we first measured the KOR mRNA expression levels and found that KOR was significantly down-regulated in HCC tumour tissues. Furthermore, we examined the correlation between KOR expression and clinicopathological parameters. Interestingly, the results exhibited that no matter in mRNA or protein level, low KOR expression was significantly associated with aggressive parameters, such as large tumour size, vascular invasion, poor differentiation and advanced TNM stage. A recent study showed that KOR-regulated lung carcinoma or melanoma invasiveness and metastasis were accompanied by changes in vascular endothelial growth factor (VEGF) [20]. The Kaplan-Meier survival analysis displayed that HCC patients in the KOR-positive group had a better OS and RFS than did those in the KOR-negative group. Unlike the principle of classification described above, we also grouped 174 patients into 3 novel groups. Consistent with our previous results, patients in the KOR gain group (KOR^T > N^) had the best outcomes among all patients, although HCC showed down-regulation of KOR expression. A multivariate analysis demonstrated that down-regulation of KOR in HCC was an independent and significant risk factor for both OS and RFS after surgery. A different report demonstrated that KOR expression in ESCC was associated with poor prognosis [19]. However, our data suggested that KOR might act as a tumour suppressor and could be a potential prognostic factor for HCC.

Numerous biomarkers of hepatocarcinogenesis have been identified in recent researches, and AFP has been recognized as the standard HCC tumour biomarker for a long time [29]. Elevated AFP levels are closely associated with HCC carcinogenesis and a high recurrence and mortality rate after hepatectomy [30]. In the current study, we classified 174 patients into 2 groups based on AFP levels with a cut-off point of 20 ng/ml. Interestingly, we found that patients in the KOR-positive group with a high AFP level exhibited better outcomes than did those in the KOR-negative group; the OS and RFS were 78 and 53% versus 35 and 25%, respectively. These results revealed that KOR overexpression in tumour tissues, even in tissues with a high AFP level, were more effective at predicting patient prognosis and supported the assumption that KOR could function as a tumour suppressor in HCC.

Clinical stage is the predominant determining factor of prognosis in patients with HCC, and the TNM stage system is one of the commonly used systems [31]. According to the TNM stage system, patients in stage I are believed to be in an early stage of HCC and to experience better outcomes after surgical resection [32]. However, patients in the same TNM stage often display various clinical outcomes, and a few patients will still have a poor prognosis. Our research demonstrated that in patients with TNM stage I, the OS and RFS rates at 5 years for KOR- positive patients were 77 and 56%, respectively, whereas the OS and RFS rates 5 years for KOR- negative patients were 44 and 25%, respectively. These results suggest that the KOR down-regulation in tumour tissues predicted poorer outcomes in patients in an early stage. Moreover, the identical correlation existed in the differentiation I/II group. In conclusion, these findings indicted that the low expression of KOR in tumour tissues could indicate a worse prognosis in early stage HCC patients, which would influence treatment decisions regarding individual clinical therapy.

Kuzumaki demonstrated that U50,488H (KOR agonist) reduced the growth of gefitinib-resistant lung cancer cells through the activation of phosphorylated-glycogen synthase kinase 3β [18]. Kohei Yamamizu reported that KOR agonists inhibited tumour angiogenesis and tumour growth by suppressing VEGF signalling in both in vivo and in vitro assays [20]. These researches suggested that KOR agonists could inhibit the growth of cancer cells through the stimulation of KOR. A recent review demonstrated that the activation of KOR could be useful for inhibiting vascular formation in cancers, and suggested that KOR could be a therapeutic target [33]. These findings have implications for the decision to use opioids of activated MOR or KOR type in cancer patients during surgery or treatment of chronic pain. Growing evidence indicates that analgesics of the MOR agonist type stimulate angiogenesis and tumour progression [34]. In contrast, the analgesics of the KOR agonist type could offer therapeutic benefits by suppressing tumour angiogenesis. Our results showed that KOR up-regulation in HCC was associated with better prognosis. Low KOR expression was associated with vascular invasion in HCC patients, which indicated that activated KOR might induce the inhibition of angiogenesis and metastasis. In addition, KOR down-regulation was related with poor tumor differentiation and advanced TNM stage, which suggested that KOR might take an important part in the HCC development and progression. We therefore inferred that KOR might be a novel potential target for therapy. Nevertheless, additional studies are required to illustrate the mechanisms that underlie the antitumour effects of KOR on HCC progression.

## Conclusions

In conclusion, our research demonstrated that KOR, which was frequently down-regulated in HCC, was significantly associated with large tumour size, increased vascular invasion, poor differentiation and advanced TNM stage. Moreover, we revealed the relevance between KOR expression in tumour recurrence and patient prognosis and suggested that KOR was an independent and significant risk factor in HCC. Taken together, KOR might be a potential tumour suppressor in HCC progression and could provide a therapeutic target for HCC treatment.

## Additional file

Additional file 1: Figure S1. KOR protein expression in HCC tissue and corresponding adjacent non-tumour tissue. (DOCX 1727 kb)

## Abbreviations

AFP: Alpha–fetoprotein
AUC: Area under curve
DOR: δ-opioid receptor
EGF: Epidermal growth factor
EMT: Epithelial mesenchymal transition
ESCC: Esophageal squamous cell carcinoma
HCC: Hepatocellular carcinoma
IHC: Immunohistochemistry
KOR: κ-opioid receptor
MOR: μ-opioid receptor
OS: Overall survival
RFS: Recurrence-free survival
ROC: Receiver operating characteristic
TNM: Tumour-node-metastasis
VEGF: Vascular endothelial growth factor
