# Supplementary material for: Down-regulation of the tumour suppressor κ-opioid receptor predicts poor prognosis in hepatocellular carcinoma patients
Source: BMC Cancer. 2017 Aug 18;17:553. doi: 10.1186/s12885-017-3541-9 (PMC5562986; doi:10.1186/s12885-017-3541-9)
Supplement: Additional file 1: Figure S1. — KOR protein expression in HCC tissue and corresponding adjacent non-tumour tissue. (DOCX 1727 kb) [file 12885_2017_3541_MOESM1_ESM.docx]

Additional file 1


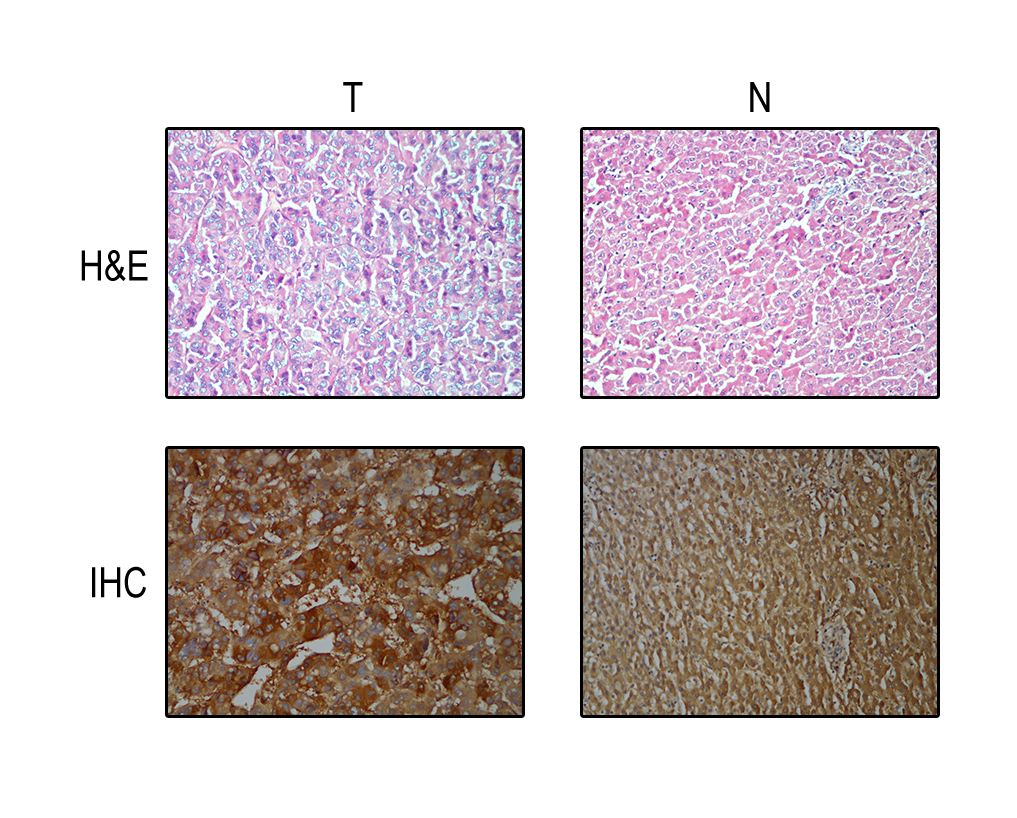


**Figure legends**

**Figure S1** KOR protein expression in HCC tissue and corresponding adjacent non-tumour tissue. (HE&IHC: magnificaton×200) The duplicated images in Figure 2, 3 and Figure S1 represent the same experiment.
